# Repair of a multi-subunit defect of the nasal ala, perialar cheek, and apical triangle of the upper lip

**DOI:** 10.1177/2050313X231193075

**Published:** 2023-08-12

**Authors:** Michelle Le, Rahul Nanda, Manish Khanna

**Affiliations:** 1 Division of Dermatology, McGill University, Montreal, QC, Canada; 2 Division of Dermatology-Oncology, Jewish General Hospital, Montreal, QC, Canada

**Keywords:** Dermatology, Mohs, surgery, reconstruction, wound

## Abstract

A 67-year-old female was treated for a left nasal ala basal cell carcinoma; post-Mohs defect was 1.5 × 1.5 cm and extended to involve the perialar cheek and apical triangle of the upper lip. How would you repair this defect?

## Discussion

Repair of surgical defects involving multiple subunits poses a major technical challenge as it requires reconstruction of all subunits involved while attempting to preserve the normal anatomic borders between them. This defect involved three cosmetic subunits consisting of the lateral nasal ala, the apical triangle of the upper cutaneous lip, and the perialar cheek at the melolabial fold. Important considerations for the repair included preservation of the free margin position of the nasal ala, lower eyelid and upper lip, proper delineation of the cosmetic subunits, restoration of the nasal ala, and recreation of the alar-facial groove and melolabial fold.

Various reconstructive options were considered for this multi-subunit defect. Healing by second intention in this area would not have been cosmetically acceptable as it would likely cause retraction of the nasal ala and possible upward retraction of the upper cutaneous lip.^1,2^ A full-thickness skin graft may have caused blunting of the alar-facial groove and the melolabial fold and would not have yielded a cosmetically acceptable result as alar skin has unique textural and color characteristics distinct from those of the nearby lip and cheek.^1,2^ Often, multiple flaps are the optimal option to recreate the nasal ala, cheek, and upper cutaneous lip while preserving the melolabial fold, alar-facial groove, and adjacent alar groove.^3^

## Resolution

**Figure 1.**
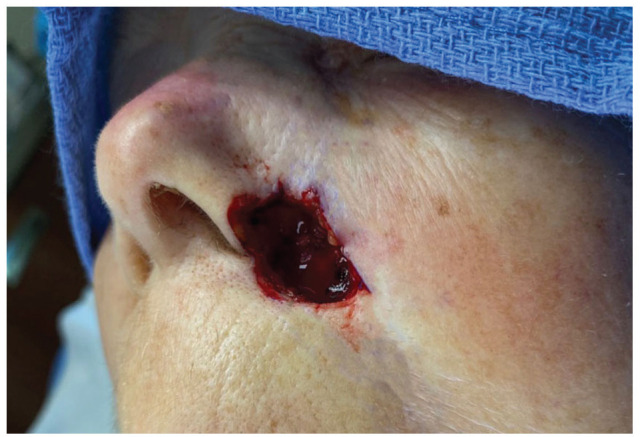
Post-Mohs surgical defect, left nasal ala, extending onto the left perialar cheek and apical triangle of the upper lip, 1.5 × 1.5 cm.

We decided to close the apical triangle defect on the upper lip with a flap from the same cosmetic unit. An island pedicle flap was incised inferiorly to the melolabial fold. This skin was advanced medially and upward to reconstruct the upper lip as a V-to-Y closure where the alar-facial groove would normally lie. The inferior aspect was closed primarily, and the flap was sutured in place with both subcutaneous absorbable monofilament suture and cutaneous nonabsorbable monofilament sutures (Figure 1). This flap closed upper lip defect, significantly reduced the size of the original defect, all while recreating the melolabial fold.

**Figure 2.**
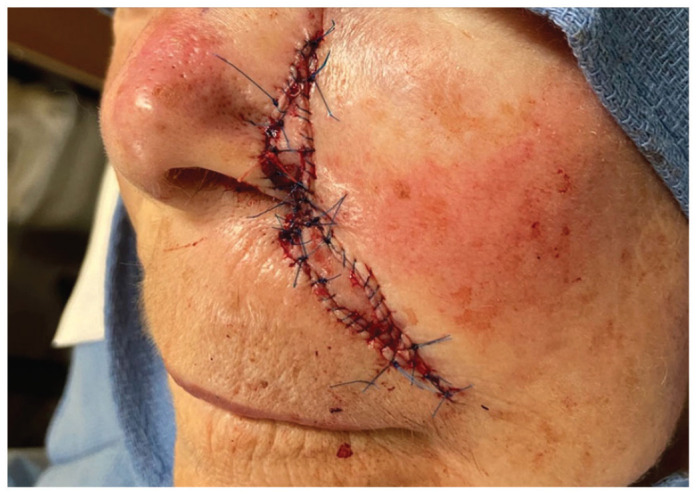
Immediate postoperative repair using double island pedicle flaps for the nasal ala, perialar cheek, and melolabial defect.

**Figure 3.**
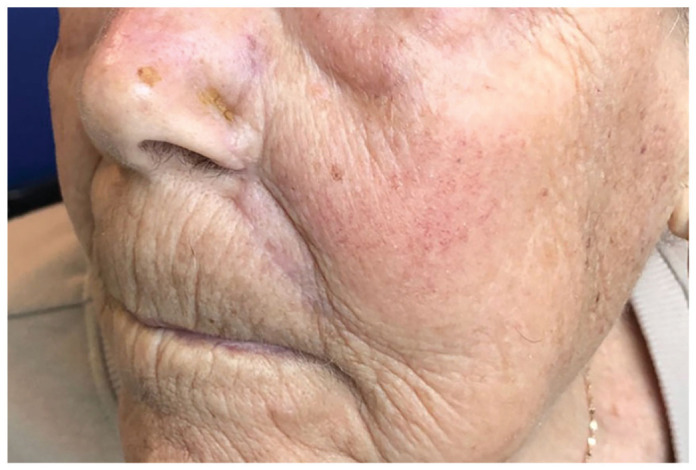
Four months postoperative, side view.

Similarly, an island pedicle flap was used to close the nasal ala and perialar cheek defects. The island pedicle flap was incised at the infraorbital cheek, lateral to the nasal sidewall, where the leading edge of the flap was advanced to the medial aspect of the alar defect and the trailing apex ran lateral to the nasal sidewall. Primary closure of the apex was accomplished without distortion of the nose. The flap was sutured in place with both subcutaneous absorbable monofilament sutures and cutaneous nonabsorbable monofilament sutures (Figure 1). This second island pedicle flap recreated the alar-facial groove at the point where the two flaps meet. The patient’s postoperative course was uncomplicated (Figure 2). Sutures were removed 2 weeks post procedure and a satisfactory cosmetic outcome was seen at 4 months (Figure 3). Although intralesional triamcinolone acetonide and resurfacing were offered to the patient to refine cosmetic results, the patient declined.

The island pedicle flap is a random-axis flap that allows for diverse reconstructive options that does not require a complicated two-staged procedure. While the nasolabial interpolation flap or paramedian forehead interpolation flap were alternative repair options, these flaps are larger, rely on a single arterial supply, and are more complicated as they require patients to undergo an additional procedure to take down the pedicle and have more extensive, complicated wound care.^4,5^ In summary, we present a double island advancement flap as a repair option for a multi-subunit defect involving the nasal ala, perialar cheek, and apical triangle of the upper lip, allowing the maintenance of the cosmetic subunits without functional repercussion.

## Conundrum keys

(1) Multi-subunit defects often require multiple flaps to recreate normal anatomic borders.
(2) Preservation of the alar-facial groove and the melolabial fold are important cosmetic considerations in defects involving the ala, cheek, and upper lip.
(3) The double island pedicle flap provides a viable repair option which allows the movement of adjacent skin for the best possible color and texture match with the ability to recreate cosmetic subunit borders.
